# Effectiveness of Play Therapy Programme in Promoting Early Child Development of under-5 Children visiting Tertiary Care Hospital in Rural Settings: Study Protocol of  a Randomized Controlled Trial.

**DOI:** 10.12688/f1000research.142194.1

**Published:** 2024-01-08

**Authors:** Manoj Patil, Abhay Gaidhane, Zahiruddin Quazi

**Affiliations:** 1 Community Medicine, Jawaharlal Nehru Medical College, Datta Meghe Institute of Higher Education and Research, Wardha, Maharashtra, 442001, India; 2 School of Epidemiology and Public Health, Community Medicine,, Jawaharlal Nehru Medical College, Datta Meghe Institute of Higher Education and Research, Wardha, India; 3 Research & Development, Community Medicine, Jawaharlal Nehru Medical College, Datta Meghe Institute of Higher Education and Research, Wardha, India

**Keywords:** Early Child Development, Play Therapy, Hospitalization, Parenting, Socioemotional, Assessment

## Abstract

**Background:**

Good care and conscious, mindful parenting are the essential requirements of the early childhood period. Good care in early childhood consists of mindful handling of children’s needs like hunger, care in sickness, socio-emotional needs, safety, love and affection. Good care promotes children’s development and provides a favourable environment for learning. It’s a well-known fact that hospitalization is a stressful situation for child of any age. Play is an integral part of early childhood period which is virtually restricted in hospital settings. This study will try to explore the effectiveness of play therapy delivered in hospital settings, on early child development.

**Methods:**

This will be a randomized control trial conducted in the Paediatrics department of a tertiary care hospital in Wardha, India. The sample size will be 360 children, 180 each in intervention and control arm. The hospitalized children from intervention arm will receive customized Play Therapy sessions from trained research assistants and will be followed up at scheduled home visits for one year from the date of enrolment. All children will be assessed for changes in cognitive, motor and socio-emotional development scores. The differences in development scores between intervention and control groups will be compared and effect sizes of changes will be calculated.

**Discussion:**

Implementation of play stimulation activities have been reported as useful strategies for promoting child development and good recovery during hospitalization. This trial will attempt to explore the effectiveness of play therapy on changes in child development scores of children from intervention group compared to the control group. The customized play therapy program will be adapted to paediatric inpatient settings, and attempts will be made to improve the Play Therapy kit for hospitalized children.

**Registration:**

Clinical Trial Registry of India Registration No.: C.T.R.I./2022/03/041355, dated 24/03/2022. Protocol Version: v5 Dated 05/09/2023.

## Introduction

The early childhood period is crucial, usually defined as the period from birth to six years of age. Good care, love, affection, addressing the needs of hunger, safety, socio-emotional issues and care during sickness etc are essential aspects of conscious parenting that promote a positive environment during childhood. A favourable and positive environment during childhood is helpful in providing children the opportunities to learn and thrive. Right from birth, children get bonded to special adults and try to learn the important basic skills by copying them. The learnings from these relationships serve as a base for preparing the children for life skills. ^1^ It was found that globally over 200 million children fail to reach their developmental potential in the first five years due to poverty, poor health services, poor nutrition, and lack of appropriate psycho-social care. ^2^ Early childhood is a period of rapid development. Play and recreation are naturally an essential part of childhood and are vital to normal development. Through various types of play and recreational activities, children learn, try to express themselves, cope with anxiety, develop their skills and master the experiences. Play can also help learn how to adapt and tolerate the healthcare and hospitalization experience. In a sense, play and recreation mimic the therapy of allowing children to explore, process, and express their healthcare experiences in a safe and protected environment. ^3^

Hospitalization is quite stressful for a child of any age. Even older children need their parents during a serious illness, and they cannot tolerate their absence even for a short period. They want their parents to be there when they need them and wish to be loved and missed. Play is an essential part of a child’s life and is key to fostering a child’s growth and development. Toys serve as the ‘tools’ of play and help to create a more ‘natural’ environment for a child. Selection and use of appropriate toys can help to reduce the traumatic effects of Hospitalization and healthcare experiences. It also aids in the speedy recovery from the illness. Play can be integral to the hospitalized child’s care and treatment plan. Play helps to support the child by offering the opportunity for creative expression, coping, and diversion. Several studies conducted in various hospitals provide evidence that a supervised play program can effectively provide a warm and child-friendly atmosphere that can help promote the child’s growth and development. In bigger hospitals and healthcare facilities, a childcare specialist can coordinate the play therapy program provided a proper play zone, suitable materials, and playmates are available. It is evident that, for any child, play is an effective way of learning, and play materials, toys, and equipment serve as the ’learning tools’. ^4^

It’s a well-known fact that play is useful in accelerating the growth and development of children. In many Western countries, play therapy alleviates the stress experienced by paediatric patients and their families during hospitalization. ^5^ Play constitutes an important parameter of a child’s normal development. Also, play serves as an important means of communication in childhood. Although, a child’s ability to play may be influenced by the child’s physical or mental disease. Play can prove to be of special therapeutic value for sick children. It can help to enhance their physical and emotional well-being. It can help to explore the issues related to the child’s experiences during hospitalization and can help to reduce the influence of negative feelings during hospitalization. Play, blended with the treatment plan, can be quite helpful in promoting the child’s growth, development, and life skills. Healthcare professionals can incorporate play as a part of treatment and care strategies for hospitalized children. The role and value of play are greater in the case of children with life-threatening diseases, disabilities, and vulnerable situations. Play therapy during Hospitalization can help to restore the child’s ability to play which was discontinued due to hospital admission. It can help to manage a child’s confidence that he can continue his/her normal life inside the hospital. With this understanding of play, healthcare professionals can explore the effects of Hospitalization and disease on children and enhance their emotional development. Through play, children can learn to gain control in different situations. Play can help to change the hospitalization experience into a positive experience. Appropriate activities must be chosen to help the children grow, encouraging the caregivers to bring the child’s favourite toys to the hospital and get actively involved in the procedure. Play promotes healing, improves coping potential, and helps tackle fears and express the children’s feelings. Hospitals should try to use play activities to reduce stress and convert the negative aspects of the hospital experiences into a positive experiences. ^6^

### Impact of hospitalisation on children

Hospitalization plays a significant effect in a child’s life. Many children admitted to hospitals have complex healthcare needs, and some require highly technical interventions. According to the 2013 statistics by WHO, children under the age of five died from diseases like acute respiratory infections, birth asphyxia, diarrhoea, neonatal sepsis, injuries, congenital anomalies, and infant prematurity. ^7^ Children hospitalized with the above diseases tend to have a traumatic experience at a hospital, such as in the long-run hospitalization tends to demonstrate negative psychological and behavioural response. ^8^ Usually, the hospitals are focused more on physical recovery and mortality than the overall well-being of the child and her family. ^9^ Children tend to perceive hospitals negatively, which impacts their physical and mental health. A study found that hospitalized children felt scared, bored, and alone. ^10^ Children experience happiness, sadness, anger, and fear in the presence of a doctor and nurse. ^11^ In another study, it was found that there was a reciprocal relationship between anxiety, blood pressure, and heart rate. ^12^ It was also found that when children were asked to draw a person in a hospital, the drawings indicated anxiety. They drew pictures of people who were sad and dependent. ^13^

Furthermore, according to the parents, a child’s behaviour, function, and health before hospitalization and after hospitalization are significantly different. Children suffer from emotional distress three to five months after hospitalization and surgery, which is most common in the age range of one year to six years. Trauma that children face at the hospital has been termed Paediatric Trauma Mental Stress which is characterized by avoidance, re-experience of the event, and hyperarousal which arises from a major illness or medical Intervention that causes a threat to a child’s health and is considered to be intrusive, painful and alarming medical care. Children tend to internalize their problems and may have symptoms of depression and anxiety which further aggravates their illness. ^14^ Certain risk factors are involved when children are hospitalized or undergo surgery. Some risk factors may be attributed to their age range, temperament, baseline anxiety, past medical encounters, and parents’ Level of anxiety. Children tend to suffer from anxiety while hospitalized due to uncertain treatment possibilities. ^15^

### Play stimulation for hospitalised children

Children, when hospitalized, tend to look for activities that can keep them calm. In some instances, they look toward their parents so that they can speak to them and look toward other children with whom they can play instead of getting bored. Some children found the hospital as a place where they can explore, learn and make new friends. ^10^ Physical exercise, healing touch, music therapy, therapeutic massage, and health education have decreased cancer symptoms. ^16^ Play activities that include art have also affected children’s overall health at the hospital. In the case of infants if their parents talk to them, massage them, make eye contact, sing to them or put objects at a closer distance and the infant grabs them, shows infants bold colours, or plays games like peek-a-boo or mimicking what the child is saying can soothe the child. ^2^

Other activities have also assisted in alleviating the symptoms of anxiety and pain. A study found that children prefer natural landscapes with calming colours over abstract paintings with bold colours. The realistic painting paved the way for positive physiological outcomes and was used for distraction, reducing pain perception. ^17^ Reading also influences children’s well-being, and when caregivers read books to children when they are sick, children feel comfortable, and their emotional affiliation with the caregivers grows. ^18^ Videoconferencing with the family has also assisted in reducing the stress of children. ^19^ Surgical procedures can create anxiety in children as well as their parents. A study found that before the children went through surgery, the nurses taught them the procedures and outcome of the surgery. The nurses, as well as the children, re-demonstrated the procedure in dolls. Since they observed it, this reduced their anxiety and caregivers. ^17^ When children above the age of four were educated about their health, they showed lower levels of anxiety than children younger than them. When play routine was introduced to hospitalized children, a lower cortisol level in their urine was indicated. ^20^ Play activities can help in reducing the organic symptoms in children. The time spent at the hospital can also be shortened when children participate in activities related to play stimulation.

### Rationale

As per the census of 2011, India had a population of 121.1 crore (Cr), among which 16.45 Cr children were in the age group of 0-6 years, and 37.24 Cr belonged to the age group of 0-14 years, which constituted around 13.59% and 30.76% of the total population, respectively. 74% of the children aged 0-6 years reside in rural areas, whereas the rural population constitutes 69% of the total population of India. ^7^ In India, the hospitalization rate of children aged 1-6 years is almost doubled due to common ailments like fever and diarrhoea. In contrast, hospitalization due to non-communicable diseases and injuries has also increased greatly over the last decade. ^8^ The average length of hospital stays ranged between 6-15 days, depending on the type of ailment. ^9^

The role and value of play are greater in the case of children with life-threatening diseases, disabilities, and vulnerable situations. Several diseases and conditions require the Hospitalization of children for weeks to a month. Play therapy during hospitalization can help to restore the child’s ability to play which was discontinued due to hospital admission. It can help to manage a child’s confidence that he can continue his/her normal life inside the hospital. With this understanding of play, healthcare professionals can explore the effects of Hospitalization and disease on children and enhance their emotional development.

Acharya Vinoba Bhave Rural Hospital (A.V.B.R.H.) in Wardha is a tertiary care hospital of Datta Meghe Institute of Medical Sciences and catering to the healthcare needs of the rural population from five districts Wardha, Nagpur, Chandrapur, Yawatmal, and Amaravati. A.V.B.R.H. is a 1,500 bedded healthcare facility with a specialized Paediatric Department, dedicated Neonatal Intensive Care Unit, and Child Development Centre. This study will focus on developing a locally adapted Play Therapy Programme for hospitalized children, the sensitization of parents regarding child development, and developing a safe, harmless, and sterilizable Play Kit for hospitalized children to minimize the risk of transmission of infection from children to children or healthcare staff. An earlier preprint version of this article can be found on Research Square. ^21^

### Objectives

*Primary objectives:*

1. To improve the motor, language, cognitive, and social-emotional development parameters of under-five years children visiting A.V.B.R.H. through play therapy and follow-up at scheduled home visits.
2. To enhance the knowledge and skills of parents on early child development and improve parent-child interactions.

*Secondary objectives:*

1. To assess the effectiveness of the dedicated Play Therapy Kit specially designed for hospitalized children, providing follow-up and assessment mechanisms through online mode.

### Trial design

This will be a randomized controlled exploratory trial. The study participants will be randomized into Intervention and Control groups with a 1:1 ratio using block randomization.

### Study setting

This study will be conducted at the Department of Paediatrics in Acharya Vinoba Bhave Rural Hospital, Wardha, and nearby villages within a 25 K.M. radius. Wardha is the smallest district in the Maharashtra State of India.

### Eligibility criteria

*Inclusion criteria:*

1. Children admitted to the Paediatric Ward of A.V.B.R.H. aged six months to 60 months.
2. Children from villages within 25 Km periphery of A.V.B.R.H.
3. Parents consenting to the participation of their child in the Play Therapy Programme.

*Exclusion criteria:*

1. Seriously sick, moribund children with life-threatening conditions.
2. Children in Intensive Care treatment.

The Investigator will take written informed consent from the parents of the hospitalized children. The comparator (control) group will include children from the adjoining paediatric ward admitted during the period when interventions will not be implemented. Children from this group will receive all required treatment and nursing care except the Play therapy sessions. This will help to avoid any contamination. Baseline assessments and socio-demographic information of these children will be collected at enrolment. Follow-up home visits and assessments will be done at six months and 12 months from the date of enrolment. If successful, the children from control group will receive play therapy after the trial is over.

**Figure 1.**
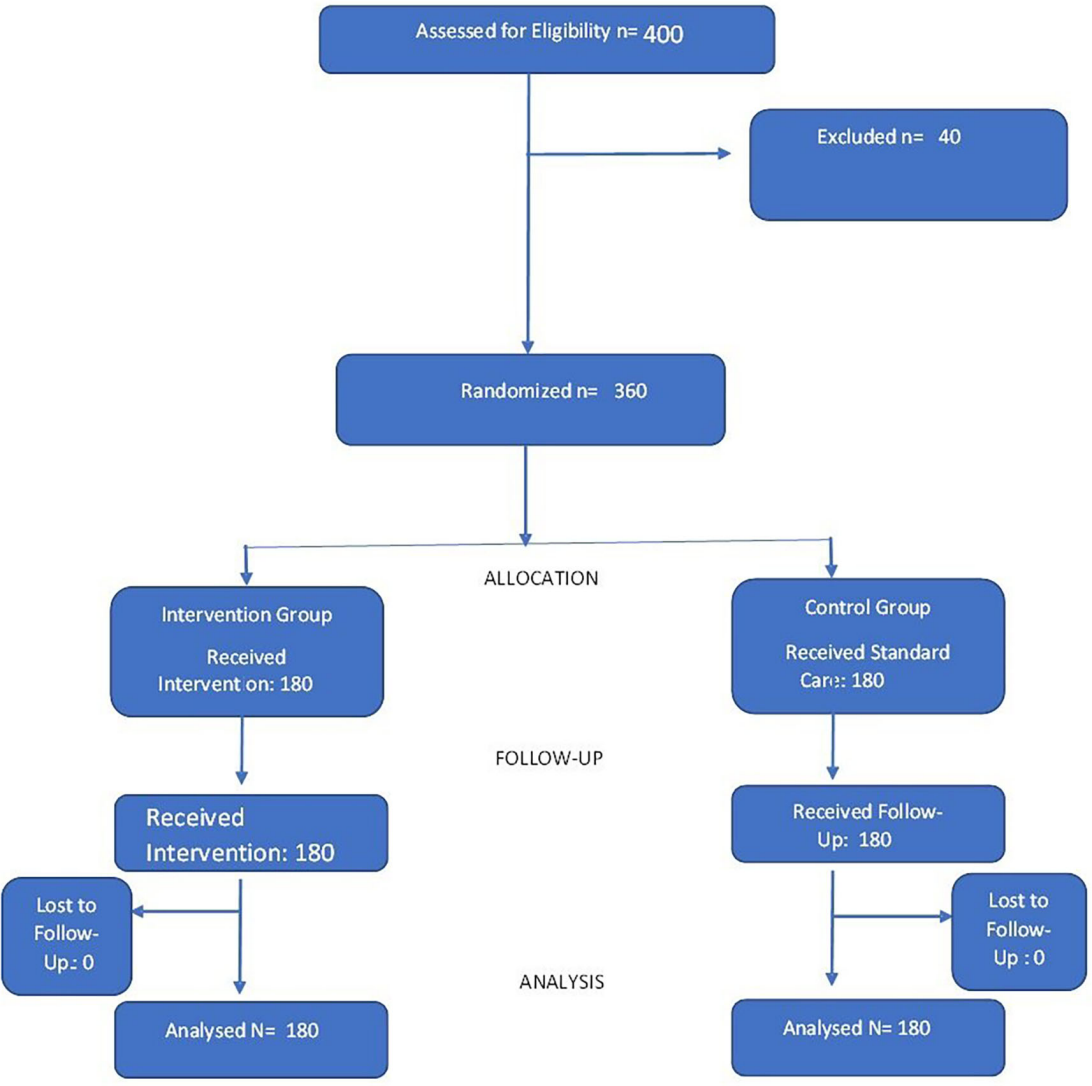
Consort flow diagram.

The children and their parents signing the informed consent will be enrolled in the study. All enrolled children hospitalized in A.V.B.R.H., scheduled to stay for a minimum of three days in the hospital, will be assigned a Unique ID. Children will be allocated to intervention or control groups using a block randomization process ^22^^,^^23^ with age groups as the blocking variable. Intervention group will receive the play therapy sessions along with the required treatment and nursing care during hospitalization stay. Sociodemographic profile and baseline assessments will be done. Play therapy sessions will be conducted at the dedicated play area in hospital. Also, these children from the intervention area will receive the play sessions at scheduled monthly home visits. Figure 1 shows the Consort Flow Diagram of study participants.

The play therapy manual has been developed by the Principal Investigator with the activities specified as per the domains of child development including physical development, language and communication skills, socio-emotional development, and cognitive development. A couple of parents and children were involved and their inputs were included in the development and piloting of the Play Therapy Manual. The Play Activity manual ^24^ is available in the extended data. ^36^

The Principal Investigator will train the team of Research Assistants on the intervention package. The trained and certified Research Assistants will deliver the intervention in hospital setting and then at home visits as per the schedule drafted by Principal Investigator.

### Duration of the interventions

1. All the children who fall in the age range of six months to five years (60 months) old will receive the intervention (along with their parents) every day (minimum three days) during their hospital stay for one hour per day.
2. The children will receive monthly follow-up sessions through home visits (or through social media group meetings if needed) for one hour every week till 12 months from enrolment in the program.
3. Activities described in the Play Therapy Manual will be followed for the follow-up sessions.

### Criteria for group sessions at the hospital

The hands of parents and their children will be washed before the activities start to avoid infectious contamination. Children will be divided into two groups as below:

1. Six months to 30 months old
2. 31 months to 60 months.

The intervention delivery details are as below:

On first day of hospitalization, parents and children will be interacted at Paediatrics ward. Randomization will be done and the parents in intervention arm will be sensitized about Play Therapy Programme. Written informed consents of parents will be taken from those who consent for participation and will be enrolled in the programme and e-learning platform/Whatsapp group.

On second day of hospitalization, a session will be conducted at designated play area in hospital. The session will emphasize on importance of early child development, its implications; diet, nutrition and healthcare of children. This session will be followed by play interaction session on cognitive and language development.

On third day of hospitalization, a play session on motor developmental activities will be conducted. This will be followed by session on socioemotional development and activities to promote it. Relevant Documents and reading materials in local language will be shared with parents during these sessions. The enrolled children will participate in play sessions and activities till the child is hospitalized.

After discharge from the hospital, parents and children will be contacted through online mode and session will be conducted through Whatapp video call/Google classroom at monthly intervals. The home visits will be scheduled for the assessment of Home Environment and Parent-Child Interactions. The online monthly sessions will be continued till one year from the date of enrolment of child in the programme. Appropriate medical and nursing care will be provided during the hospital stay. If needed, referral services will be provided during follow-up at-home visits. If Children and parents insist on discontinuing play therapy, the sessions will be discontinued for those children.

**Figure 2.**
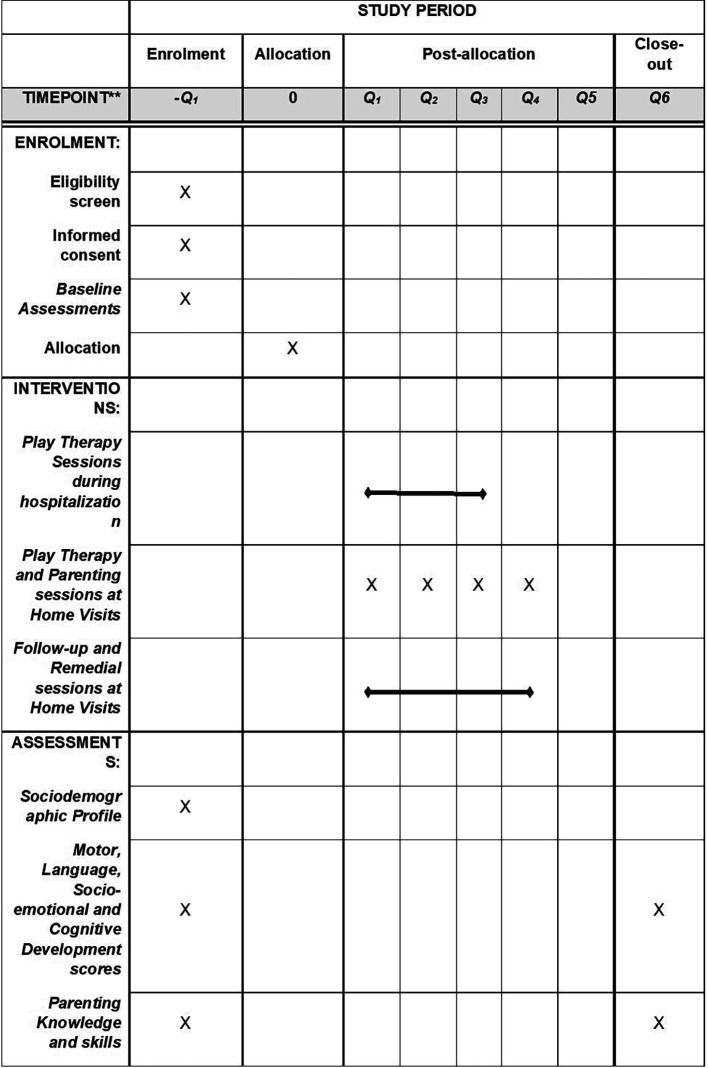
The schedule of enrolment, interventions, and assessments.

After one-year of enrollment, assessments of age-appropriate developmental milestones of children will be done either in the hospital ECD Centre or through personalized home visit to the enrolled family. Age-appropriate assessment tools will be used. Reports will be shared with parents, and follow-up instructions and meeting schedules will be shared. Figure 2 shows the schedule of enrolment, interventions, and assessments.

There is no anticipated harm from the trial participation. The study participants will be provided with the printed copies of Play Therapy Manuals after completion of trial. The children with below average development scores will receive additional ECD services at hospital ECD centre and will receive follow-up till next one year through home visits.

### Outcomes

*Primary outcomes:*

1. Improvements in mean scores of child developmental parameters for cognitive, physical, socio-emotional, and language development by the end of one year of intervention.
2. Improvements in knowledge and skills of parents regarding early child development by the end of one year of intervention.

*Secondary outcomes:*

1. Improvements in the parent-child satisfaction levels regarding the in-hospitalization care and follow-up by the end of one year of intervention.

Figure 2 details the schedule of enrolment, interventions, and assessments.

### Sample size

With reference to the study conducted by Gaidhane *et al.,* ^25^^,^^26^ the expected difference in child development outcomes between the intervention and control groups is 0.5 SD, considering a dropout rate of 10%, level of significance = 5%, and power = 80%. The formula for calculating sample size for two independent means is:

$$n=\frac{2\;{\left(\mathrm{Z\alpha}+\mathrm{Z\beta}\right)}^{2}\times \delta^{2}}{d^{2}}$$

where

n = Minimum sample size required in each group,

d = Expected mean difference between two groups = 0.5

δ = standard deviation = 1.69 (As per Previous Study)

Zα: value of type I error at 5% significance level =1.96

Zβ: value of type II error at 80 % Power (1-β) = 0.84

Based on the above formula, the calculations are as follows:

$$\begin{array}{c} n=\frac{2\;{\left(1.96+0.84\right)}^{2}\times 1.69^{2}}{{\left(0.5\right)}^{2}}\\ =\frac{2\left(7.84\right)\times 2.86}{0.25}\\ =\frac{44.84}{0.25}=179.37\approx 180 \end{array}$$

Thus, the sample size required per group is 180. Hence total sample size required is 360. A sample size of 360 subjects, 180 in each arm, is sufficient to detect a difference of 0.5 between groups in developmental scores, assuming a standard deviation of 1.69 using a two-tailed t-test of difference between means with 80% power and a 5% level of significance. For assessing the changes in parenting knowledge and skills, parents of all children in intervention group **(180)** will be assessed.

### Recruitment

Parents of children reporting to A.V.B.R.H. in the first three months (January-March 2023) will be enrolled in the trial. All participants consenting to participate in the study will be assigned the Unique ID. Using a blocked randomization process with age groups as the Blocking variable, participants will be allocated to intervention or control groups. Age groups will be:

1. Six months to 30 months
2. 31 months to 60 months.

All children hospitalized in A.V.B.R.H., scheduled to stay for a minimum of three days in the hospital, and their parents signed the informed consent will be enrolled in the study.

### Assignment of interventions: allocation

All participants consenting to participate in the study will be assigned the Unique ID. Sequence generation will be done using computer generated random numbers. Using a block randomization process ^10^^,^^11^ with age groups as the blocking variable, participants will be allocated either to intervention or control groups.

Sequentially numbered, opaque, sealed envelopes will be used for implementing the allocation sequence.

The Principal Investigator will generate the allocation sequence, trained assistants will enroll the participants, and assign participants to intervention and control groups. The Principal Investigator will train the Research Assistants for intervention, and assessors for baseline-endline assessments, supervise the data collection process and monitor the trial implementation process. We will ensure that all adults associated with the trial have the appropriate permissions and training and criminal records check to work with children. The outcome assessors and data analysts will be blinded.

### Data collection and management

Data will be collected in pre-tested O.D.K. based app with inbuilt checks for data quality, identifying duplicate entries. The data received on dedicated server of Research Cell of Datta Meghe Institute of Medical Sciences will be imported and checked on real time basis. The data will be encrypted for sensitive information and will be handled by the assigned team including Information Technology Coordinator and statistician. The datasets will be shared and made reusable according to FAIR data principles.

Assessments will be conducted using the tools as detailed below:

1. Physical Development (Using Developmental Milestones Checklist-II) ^27^^,^^28^
  a. Improvement in Fine Motor scores
  b. Improvement in Gross Motor scores
2. Cognitive Development Scores (Using Developmental Milestones Checklist-II)
3. Language Development Scores (Using Developmental Milestones Checklist-II)
4. Socioemotional Development Scores (Using Profile of Socio-emotional Development) ^29^
5. Home Environment (Using HOME Inventory Assessment) ^30^^–^^32^
6. Parent-child Interactions (Using Observation of Mother-Child Interaction Tool) ^33^

The research assistants will be trained on the use of all these tools and inter-rater, intra-rater scoring exercises will be conducted during training. To promote participant retention and complete follow-up, all enrolled children in the intervention group will receive scheduled home visits at monthly intervals. The children in the control group will be followed up telephonically at monthly intervals. Also, telephonic follow-up will be taken for those missing home visits in intervention group. The outcome data will detail the participants who discontinue or deviate from intervention protocols.

### Data management

All data will be collected in Tablet P.C. based O.D.K. collect app with an in-built range check, double entry, and value checks. Data will be exported to the server after ensuring the correctness of the data entered. The data from the server can be downloaded as an xls file. Each participant will be allotted a Unique ID which will be used for analysis and reporting purposes. All data will be anonymous and personal information will be maintained to protect confidentiality before, during, and after the trial.

### Statistical methods

After appropriate cleaning and compilation, the pre- and post-intervention child development data in specified domains will be collected and fed to STATA-14 by the assigned statistician. Individual and child age-group wise scores of developments will be calculated and tabulated. Mean differences in child development scores for intervention and control groups and pre-post intervention will be calculated. The effect size by Cohen’s-D will be calculated for each child age group, and significance of differences in effect size will be estimated. Also, qualitative subgroup and adjusted analyses, Difference in difference analysis for motor, language, cognitive and socio-emotional development scores will be done. Attempts will be undertaken to ensure the collection of complete data. In cases of missing data, families will be contacted through home visits and missing data will be collected. If any case/family could not be tracked, iterative algorithm or multiple imputation technique will be used to handle the missing data. The datasets analysed during the current study, statistical code and full protocol will be made available by publishing on open data repository Zenodo.

### Oversight and monitoring

*Composition of the coordinating center and trial steering committee*

The Trial Steering Committee (TSC) consist of the following:

1. The Head of the department of Public Health
2. PhD Supervisor
3. Director, Research and Development
4. The Head of the department of Pediatrics
5. Principal investigator
6. Statistician
7. Data manager

The Principal Investigator is responsible for all aspects of local organisation including identifying potential recruits and taking consent. The PhD Supervisor, Head of Public Health Department, Director Research and Development, and Head of Paediatrics department will be supervising the trial. Trial Steering Committee (TSC) will meet over the course of the trial to oversee conduct and progress in monthly meeting.

*Composition of the data monitoring committee, its role, and reporting structure*

The data monitoring committee (DMC) consists of Research-in-charge of the institute and Research-in-charge and Data Manager of the department. All related data will be managed and monitored by Principal Investigator, along with support from a statistical expert as per the guidance of Data Monitoring Committee. Since this is a play therapy intervention, hardly any adverse events and other unintended effects of trial interventions or trial conduct are expected. If encountered, those will be reported per the guidelines from the Institutional Ethics Committee. Project Management Group will review the trial conduct in monthly meetings. The Trial Steering Group and the independent Data Monitoring and Ethics Committee will review the conduct and progress of trial in quarterly meetings. Important protocol modifications will be communicated to relevant parties (e.g., investigators, R.E.C./IRBs, trial participants, and trial registries through emails and printed Hard copies, as required).

### Dissemination plans

The project results and data will be published on Stepping Stones Project website of our Institute. ^34^ The trial results will be published in Scopus, Pubmed, and Web of Science-indexed specialty journals on early child development and have access to healthcare professionals, the public, and other relevant groups. The participants will be informed of the results through personalized visits and provided the information brochures.

## Discussion

When a child is engaged in play activities it plays an important role in decreasing the stress of parents and children. As the studies suggest, play stimulation can play an important role in relieving the symptoms of stress and organic diseases. The hospital staff needs to be trained; play stimulation should occur in the wards, and counselling should be given to the parents and their children to release their stress. Before the play stimulation activities should be practiced, the caregivers, therapists, hospital staff, and parents must understand what is expected in different age domains related to cognitive and psychosocial development.

This [lay therapy intervention package for hospitalized children will be tested for its effectiveness in the Acharya Vinoba Bhave Rural Hospital, Wardha, India. The data of changes in child development parameters will be analysed. If this trial is successful to bring out positive changes in child development parameters of intervention group compared to control group, it can prove to be an evidence-based proof of concept for the future ’Play Therapy Stimulation Package’ for hospitalized children. If the trial fails to bring-out improvements in child development parameters in intervention arm compared to control arm, the gaps and deficiencies will be explored and attempts will be done to reframe the trial for reimplementation. As this will be a single centre study, the response rate and acceptance of intervention by beneficiaries may need to be generalizable to different types/levels of inpatient childcare facilities. A minimum of three days hospital stay is expected with the initial play therapy start. Staying for less time than this duration may not effectively stimulate children and sensitize parents. Also, follow-up after discharge may pose some challenges.

### Trial status

This trial was registered prospectively with Clinical Trial Registry of India (CTRI). ^35^ Recruitment of Participants started from Jan 2023. Expected date of trial completion is 30 ^th^ May 2024.

## Data Availability

No data are associated with this article. Zenodo: File Name: Activity CARDS V5_final TTS v1.pdf. Description: Activity Cards for Play Therapy Sessions. https://doi.org/10.5281/zenodo.8127629. ^36^ This project contains the following extended data:
• Activity CARDS V5_final TTS v1.pdf. (This data contains the details of different types of activities for promoting cognitive, motor, language and socio-emotional development of children). Zenodo: SPIRIT Checklist for ‘Effectiveness of Play Therapy Programme in Promoting Early Child Development of under-5 Children visiting Tertiary Care Hospital in Rural Settings: Study Protocol of a Randomized Controlled Trial’. https://doi.org/10.5281/zenodo.8318964. ^37^ Data are available under the terms of the Creative Commons Attribution 4.0 International license (CC-BY 4.0).
