# Supplementary material for: Effectiveness of Play Therapy Programme in Promoting Early Child Development (ECD) of under-5 Children visiting Tertiary Care Hospital in Rural Settings: Study Protocol of  a Randomized Controlled Trial
Source: F1000Res. 2025 Sep 19;13:28. Originally published 2024 Jan 8. [Version 3] doi: 10.12688/f1000research.142194.3 (PMC12521891; doi:10.12688/f1000research.142194.3)
Supplement: Peer review history

# Reviewer report: Reviewer response for version 3

**DOI:** 10.5256/f1000research.188294.r419662

**Published:** 2025-11-19

**Authors:** S. Alexa McDorman

**Affiliations:** 1 University of Maryland,, College Park,, Maryland, USA

This protocol describes the procedures for a randomized controlled trial of a play therapy programme for parents and their hospitalized under-5 children in rural India. The intervention provides child development guidance for parents along with interactive play sessions where parents can implement the tips they received, which has been shown to be an effective model for family learning and outcomes. Through families with young, hospitalized children in a rural setting, the study serves a population with significant healthcare needs and extensive opportunity for positive changes to cascade and increase many times over, including across the lifetimes of participating children and across families, with parents potentially sharing knowledge with other parents and putting it to use for other children. This is a valuable and thoughtfully designed study.

 From my view, this promising study needs only a few revisions for clarification, detailed below in approximate order of appearance in the manuscript. In addition, I would like to note that the Statistical Methods section is not very specific, but this is not necessarily a problem as long as the analytical process is described in full for future publications.

1. The second primary objective, focused on parents (p. 6), is left out in parts of the text, including the Abstract, Statistical Methods, and Discussion. The Data Collection and Management section mentions home environment and parent-child interactions, but not the parent skills and knowledge described in the primary object. The HOME inventory arguably measures parenting skills, but if this is the goal, it should be made clearer. 
2. The last two sentences of the first paragraph of the introduction (p. 4) need slight revision to make them understandable. I think “Play can also help learn how to adapt…” means to say “help children learn” or “help teach children” instead. I am not sure what this means: “Play and recreation mimic the therapy of allowing…” Maybe “Play and recreation are naturally therapeutic in that they allow…”?
3. Citation 16 (p. 5) is incorrect. It is attached to this statement: “Physical exercise, healing touch, music therapy, therapeutic massage, and health education have decreased cancer symptoms.” It is linked to “THE SINO-BRAZILIAN PRINCIPLES IN A LATIN AMERICAN” (References, p. 14), which is completely unrelated – it’s on legal frameworks, no mention of the relevant topics.
4. There are two secondary objectives (p. 6), but only one secondary outcome (p. 10). I recommend the authors either state why or add a clear outcome for the second secondary objective, like comparing effectiveness or satisfaction with online follow-up versus home visits.
5. Exclusion criteria on p. 6 state “Seriously sick, moribund children with life-threatening conditions.” Some discretion may be necessary, but this should be a bit more defined, if possible. Does the research team have an operational definition? (e.g., if the child is bedbound and/or highly immunologically compromised). If this is not possible, the team should track specific reasons children were excluded so that examples can be provided when non-identifiable (i.e., mentioning specific, extremely rare conditions would not be appropriate).
6. There is a typo on p. 8 – “Duration of the interventions”, point 2 says “monthly follow-up sessions. . .for one hour every week. . .” Everywhere but here says monthly, so I think “every week” just needs to be removed.
7. Although the timeline mentioned suggests that data collection may have already started, I am a bit concerned about the apparent inclusion of participants with infectious illnesses. It is good that the research team is requiring hand washing, but that does not seem sufficient to mitigate all risk, particularly with such young children, who are unlikely to have good hygiene practices. Therefore, I believe that the statement “There is no anticipated harm from the trial participation” (p. 8) and consent forms should mention that participation may increase the risk of infectious illness.

Is the study design appropriate for the research question?

Yes

Is the rationale for, and objectives of, the study clearly described?

Yes

Are sufficient details of the methods provided to allow replication by others?

Yes

Are the datasets clearly presented in a useable and accessible format?

Yes

Reviewer Expertise:

NA

I confirm that I have read this submission and believe that I have an appropriate level of expertise to confirm that it is of an acceptable scientific standard, however I have significant reservations, as outlined above.

# Reviewer report: Reviewer response for version 3

**DOI:** 10.5256/f1000research.188294.r415484

**Published:** 2025-10-14

**Authors:** Mphelekedzeni Caroline Mulaudzi

**Affiliations:** 1 University of Witwater'srand, Johannesburg, South Africa

The researchers have addressed my comments.

 I still believe that the manuscript would benefit from proofreading and grammatical edits before publication.

Is the study design appropriate for the research question?

Yes

Is the rationale for, and objectives of, the study clearly described?

Yes

Are sufficient details of the methods provided to allow replication by others?

Partly

Are the datasets clearly presented in a useable and accessible format?

Not applicable

Reviewer Expertise:

Child health; Child Development

I confirm that I have read this submission and believe that I have an appropriate level of expertise to confirm that it is of an acceptable scientific standard.

# Response

**Published:** 2025-10-14

**Authors:** MANOJ PATIL

**Affiliations:** Research and Development, Datta Meghe Institute of Higher Education and Research, Wardha, Wardha, Maharashtra, India

Dear Sir,

 Thank you very much for your timely response and approval of the changes done so for. I will revise the manuscript for minor grammatical errors as suggested and upload the final version shortly.

# Reviewer report: Reviewer response for version 2

**DOI:** 10.5256/f1000research.173000.r409412

**Published:** 2025-09-15

**Authors:** Mphelekedzeni Caroline Mulaudzi

**Affiliations:** 1 University of Witwater'srand, Johannesburg, South Africa

I commend the researcher and the team to undertake the randomized trial.

**Objectives**

 The secondary objectives appear to be two in one sentence

 Objective 1. Are they visiting or admitted to the hospital?

**Methodology**

 The methodology is not clear to me as a reader. It is not clear when individual or group play sessions will be held.

 The block grouping is too wide for the under-3 group because of rapid development in this age group. 

 Without the understanding of the ward structure, i am struggling to understand how the mothers who do not receive the intervention will be protected from knowing they did not receive the intervention, and in the long run, avoid the non-intervention study, abandoning the study. How are the wards chosen?

 There is mention in the text of scheduled home visits and in some cases exchanged with online. The researchers should edit the test such that there is consistency

 Duration of intervention 2. Is it weekly or monthly? 

 Intervention delivery details paragraph that start with on the third day. At the end of the paragraph, till the child is hospitalized or discharged

 Academic writing 

 There is a lot of repetition in the introduction.

 Sentence construction is poor, and at times, ideas are all over. The researchers may benefit from the editing services.

 Referencing in the text jumps from 24 to 36 and then back to 25 in the next paragraph.

 Abbreviations are sometimes used without initially stating the full phrase.

 It may be common to describe the population in crore in India, but it is not globally. I suggest the researchers use the common standards.

**Some specific text comments**

 "The early childhood period is crucial, usually defined as the period from birth to six years of age," I thought it was up to 8 years?

 Maybe the last heading should be conclusions

Is the study design appropriate for the research question?

Yes

Is the rationale for, and objectives of, the study clearly described?

Yes

Are sufficient details of the methods provided to allow replication by others?

Partly

Are the datasets clearly presented in a useable and accessible format?

Not applicable

Reviewer Expertise:

Child health; Child Development

I confirm that I have read this submission and believe that I have an appropriate level of expertise to confirm that it is of an acceptable scientific standard, however I have significant reservations, as outlined above.

# Response

**Published:** 2025-09-16

**Authors:** MANOJ PATIL

**Affiliations:** Research and Development, Datta Meghe Institute of Higher Education and Research, Wardha, Wardha, Maharashtra, India

**Respected Sir,**

**Mr.  Mphelekedzeni Caroline Mulaudzi, University of Witwater'srand, Johannesburg, South Africa.**

 I am extremely thankful for your precious time spent on reviewing my manuscript and your precious comments on different aspects of this file. I have tried to revise my manuscript as per your suggestions. Please see below the reply to your comments.

**I commend the researcher and the team to undertake the randomized trial.**

**Reply:** Respected Sir, Thank you very much for your appreciation.

**Objectives**

**The secondary objectives appear to be two in one sentence**

**Reply:** Correction done and two secondary objectives noted.

**Objective 1. Are they visiting or admitted to the hospital?**

**Reply: **Corrected as Children admitted in AVBRH Hospital.

**Methodology**

**The methodology is not clear to me as a reader. It is not clear when individual or group play sessions will be held.**

**Reply:**  Group Play sessions will be conducted for children in intervention arm. These sessions will be conducted for one hour every day till the child’s discharge from the hospital.

**The block grouping is too wide for the under-3 group because of rapid development in this age group.**

**Reply: **We sincerely appreciate your insightful observation regarding the block grouping for the under-3 age group. We fully acknowledge the rapid developmental changes that occur during this period. However, our tertiary care rural hospital has a great turnover of patients. We have two big Pediatric wards of nearly 100 beds each and a dedicated ICU for children. Also, the proposed intervention of play therapy will be for admitted cases in intervention arm till the duration of their stay at hospital (approx. 5 to 10 days). After that, they will be followed-up through monthly home visits till next one year. So, Block randomization seems feasible.

**Without the understanding of the ward structure, i am struggling to understand how the mothers who do not receive the intervention will be protected from knowing they did not receive the intervention, and in the long run, avoid the non-intervention study, abandoning the study. How are the wards chosen?**

**Reply: **Our hospital has 2 pediatric wards at 2 different floors 2 ^nd^ floor and 3 ^rd^ floor. The comparator (control) group will include children from the pediatric ward on another floor and admitted during the period when interventions will not be implemented for intervention group. Also, the Play sessions will be conducted at dedicated play area adjoining the selected ward. That area will not be accessible for children admitted in another ward or their parents.

**There is mention in the text of scheduled home visits and in some cases exchanged with online. The researchers should edit the text such that there is consistency.**

**Reply: **Corrected as suggested. Monthly home visits will be provided to all children in intervention arm after discharge till one year. In case the home visits are not possible, the parents will be reached through online mode (whats app/google meet) and follow-up visits will be ensured.

**Duration of intervention 2. Is it weekly or monthly?**

**Reply: **Sir, the Play therapy sessions will be delivered **daily** during child’s stay at hospital till the child gets discharged from hospital. The time of session will be one hour each. After discharge from the child will receive **monthly** follow-up sessions at home visits till next one year.

**Intervention delivery details paragraph that start with on the third day. At the end of the paragraph, till the child is hospitalized or discharged.**

**Reply: **The Play therapy sessions will be delivered from the day the patient is allotted to intervention group and his parent consents for it. It will be mostly 3 ^rd^ day of patient’s admission to hospital. These play therapy group sessions will be of one hour daily and will continue till the child remains admitted in hospital. After discharge, the family will receive home visits at monthly intervals.

**Academic writing** 

 There is a lot of repetition in the introduction. Sentence construction is poor, and at times, ideas are all over. The researchers may benefit from the editing services.

**Reply: **Thanks for the suggestion. We have edited the introduction section using Grammarly and corrected all grammatical mistakes accordingly. Also tried to reduce most of the repetitions as suggested.

**Referencing in the text jumps from 24 to 36 and then back to 25 in the next paragraph.**

**Reply: **Thanks for notifying. Reference 36 was wrongly cited in between. I have removed it and kept at last where its applicable. Now all citations areas per the order of appearance.

**Abbreviations are sometimes used without initially stating the full phrase.**

**Reply: **Thanks for pointing out. We have revised and added the full forms of abbreviations where needed.

**It may be common to describe the population in crore in India, but it is not globally. I suggest the researchers use the common standards.**

**Reply: **Thanks for pointing out. We have updated the figures in millions.

**Some specific text comments**

**"The early childhood period is crucial, usually defined as the period from birth to six years of age," I thought it was up to 8 years?**

**Reply: **Yes Sir. Thanks for notifying. It’s actually period from birth to 8 years. I have corrected in the text.

**Maybe the last heading should be conclusions**

**Reply:** Sorry Sir. Conclusion not added as the results are not available. Being a Study Protocol, the Conclusion head was omitted.

# Reviewer report: Reviewer response for version 2

**DOI:** 10.5256/f1000research.173000.r330861

**Published:** 2024-10-18

**Authors:** Sylvia Fernandez Rao

**Affiliations:** 1 ICMR National Institute of Nutrition, Hyderabad, India

This is an important article as it investigates the impact of a play intervention in a hospital setting. Previous studies have shown that long-term hospitalisation can lead to developmental losses, making this trial particularly timely, as few studies focus on this critical aspect. However, certain sections of the manuscript required clarification.

 Firstly, the CONSORT diagram indicated that the trial had been completed (covering recruitment, refusal, intervention, loss to follow-up, etc.), but the text suggested that the trial was yet to begin. This discrepancy has since been addressed by the authors.

 Secondly, it was important to clarify whether the intervention personnel would interact with the control group, as this could lead to contamination. The authors have confirmed that the intervention team will not be interacting with the control group. Additionally, the intervention was described as including nutrition and health components, raising the question of how these aspects would be distinguished from the developmental intervention involving play. The authors have clarified that the nutrition and health aspects will involve "sensitisation of parents during the hospital stay and home visits."

 I would suggest that these sensitisation messages on nutrition and health be provided to the control group as well, ensuring that the play intervention remains the exclusive component in the intervention group. With the additional details regarding the intervention design and implementation, the manuscript now provides a clearer and more comprehensive account.

Is the study design appropriate for the research question?

Partly

Is the rationale for, and objectives of, the study clearly described?

Yes

Are sufficient details of the methods provided to allow replication by others?

Partly

Are the datasets clearly presented in a useable and accessible format?

Not applicable

Reviewer Expertise:

Early child development and Nutrition

I confirm that I have read this submission and believe that I have an appropriate level of expertise to confirm that it is of an acceptable scientific standard.

# Reviewer report: Reviewer response for version 1

**DOI:** 10.5256/f1000research.155705.r260212

**Published:** 2024-09-03

**Authors:** Sylvia Fernandez Rao

**Affiliations:** 1 ICMR National Institute of Nutrition, Hyderabad, India

This is an important article as it is testing the impact of play intervention in the hospital setting. Earlier studies have shown that long term hospitalization does cause developmental loss. So this trial is timely, as not all studies focus on this aspect. However  some aspects of the manuscript need clarity. For one, the consort diagram shows that the trial is done (recruitment, refusal, intervention, loss to follow-up, etc.)  but the text gives the impression that the trial is yet to start. This needs to be clarified, and the appropriate tense should be used. 

 The intervention personnel will they be interacting with the control arm? this can cause ? Further, the intervention includes nutrition and health aspects. How will this be separated from developmental intervention of play? Will the control group receive messages on health and nutrition? A fewer details regarding the intervention would be useful .

Is the study design appropriate for the research question?

Partly

Is the rationale for, and objectives of, the study clearly described?

Yes

Are sufficient details of the methods provided to allow replication by others?

Partly

Are the datasets clearly presented in a useable and accessible format?

Not applicable

Reviewer Expertise:

Nutrition  and Early child development

I confirm that I have read this submission and believe that I have an appropriate level of expertise to confirm that it is of an acceptable scientific standard, however I have significant reservations, as outlined above.

# Response

**Published:** 2024-10-04

**Authors:** MANOJ PATIL

**Affiliations:** Research and Development, Datta Meghe Institute of Higher Education and Research, Wardha, Wardha, Maharashtra, India

Respected Reviewer,

 Thank you very much for taking the time to review this protocol and thorough assessment. Your comments and suggestions are very useful. 

 Regarding the tense in the consort diagram, I will update the diagram in version 2 of this article as suggested, and upload it shortly.  

 The intervention personnel will not interact with the control arm participants. Only assessors will contact the participants in control arm for baseline and endline assessments. Necessary steps will be taken to avoid the contamination.

 Further, the intervention includes only sensitization of parents on nutrition and health aspects during hospital stay and home visits. There is no direct nutritional intervention.

 The control group will not receive messages on health and nutrition or any other aspects of intervention. It will be a separate group from another hospital of same Institute.

 I will add the requested details in the updated version of this protocol shortly. Thank you very much for your kind perusal.
